# Liver Semantic Segmentation Method Based on Multi-Channel Feature Extraction and Cross Fusion

**DOI:** 10.3390/bioengineering12060636

**Published:** 2025-06-11

**Authors:** Chenghao Zhang, Lingfei Wang, Chunyu Zhang, Yu Zhang, Peng Wang, Jin Li

**Affiliations:** 1 College of Intelligent Systems Science and Engineering, Harbin Engineering University, Harbin 150001, China; 910381219@hrbeu.edu.cn (C.Z.); wanglingfei@hrbeu.edu.cn (L.W.); zhangyu04@hrbeu.edu.cn (Y.Z.); 2 College of Computer and Control Engineering, Qiqihar University, Qiqihar 161006, China; 03813@qqhru.edu.cn; 3 Artificial Intelligence Energy Research Institute, Northeast Petroleum University, Daqing 163318, China

**Keywords:** liver segmentation, feature extraction, feature fusion

## Abstract

Semantic segmentation plays a critical role in medical image analysis, offering indispensable information for the diagnosis and treatment planning of liver diseases. However, due to the complex anatomical structure of the liver and significant inter-patient variability, the current methods exhibit notable limitations in feature extraction and fusion, which pose a major challenge to achieving accurate liver segmentation. To address these challenges, this study proposes an improved U-Net-based liver semantic segmentation method that enhances segmentation performance through optimized feature extraction and fusion mechanisms. Firstly, a multi-scale input strategy is employed to account for the variability in liver features at different scales. A multi-scale convolutional attention (MSCA) mechanism is integrated into the encoder to aggregate multi-scale information and improve feature representation. Secondly, an atrous spatial pyramid pooling (ASPP) module is incorporated into the bottleneck layer to capture features at various receptive fields using dilated convolutions, while global pooling is applied to enhance the acquisition of contextual information and ensure efficient feature transmission. Furthermore, a Channel Transformer module replaces the traditional skip connections to strengthen the interaction and fusion between encoder and decoder features, thereby reducing the semantic gap. The effectiveness of this method was validated on integrated public datasets, achieving an Intersection over Union (IoU) of 0.9315 for liver segmentation tasks, outperforming other mainstream approaches. This provides a novel solution for precise liver image segmentation and holds significant clinical value for liver disease diagnosis and treatment.

## 1. Introduction

The liver, as the largest and one of the most functionally complex solid organs in the human body, plays a vital role in maintaining homeostasis through multiple essential functions such as metabolism, detoxification of harmful substances, immune regulation, and endocrine control [1]. Disruptions in liver function often lead to systemic imbalances involving multiple organ systems, resulting in a range of pathological conditions from metabolic disorders to systemic diseases. Consequently, systematic evaluation and early intervention for liver health remain critical priorities in clinical medicine [2].

Computed tomography (CT) is widely used in liver disease screening and diagnosis due to its high spatial resolution, rapid acquisition speed, and clear depiction of anatomical structures, enabling the early detection and therapeutic assessment of various hepatic conditions [3]. In recent years, advances in biosensor technologies—including serum biomarker assays, high-sensitivity fluorescent probes, and volatile organic compound (VOC) analysis—have provided molecular and pathological data beyond conventional imaging [4]. When integrated with artificial intelligence algorithms, these multimodal data sources can be fused through deep learning models to enhance the accuracy and efficiency of auxiliary diagnosis and disease classification [5]. Table 1 summarizes representative liver disease detection methods that combine CT imaging and biosensor technologies, alongside their corresponding AI system applications.

Although these liver diseases exhibit varying imaging characteristics, accurate liver segmentation remains a fundamental prerequisite for subsequent tasks such as lesion detection, organ measurement, tumor quantification, and liver volume assessment, regardless of whether conventional image processing or deep learning-based algorithms are used [11]. In particular, volumetric information plays a critical role in clinical scenarios such as hepatocellular carcinoma, intrahepatic cholangiocarcinoma, liver metastases, and liver transplantation, aiding in the evaluation of tumor burden, prediction of postoperative liver function reserve, and monitoring of graft recovery [12,13].

Liver image segmentation aims to accurately delineate anatomical boundaries of the liver from tomographic scans, assisting clinicians in assessing hepatic structures, localizing lesions, and analyzing their spatial relationships with adjacent vasculature. This enhances both diagnostic accuracy and the personalization of treatment planning [14,15,16,17]. The accuracy of liver medical image segmentation has markedly improved with advancements in deep learning techniques [18]. However, the complex anatomy of the liver, which includes multiple tissue types and extensive continuity with adjacent organs of similar intensity, makes CT-based liver image segmentation a particularly challenging task [19]. Additionally, CT imaging is susceptible to various sources of degradation such as blurring, inhomogeneity, and low resolution, which further complicate the segmentation process, especially for deep learning-based models [20]. Consequently, leveraging advanced image processing techniques and deep learning algorithms to address these challenges and achieve accurate liver segmentation and volumetric quantification has become a critical research focus.

Currently, automatic liver segmentation techniques can be broadly categorized into two types: traditional methods and deep learning-based approaches. Traditional approaches—such as level sets [21,22], thresholding [23], and region growing [24,25]—have demonstrated certain utility but are constrained by their reliance on manually predefined parameters. These methods typically focus on grayscale intensity, often neglecting the morphological complexity and spatial variability of the liver. In contrast, deep learning-based segmentation methods offer greater intelligence and flexibility. These approaches can be further classified into semantic segmentation and instance segmentation. Semantic segmentation aims to partition an image into regions representing different semantic categories by assigning a class label to each pixel, emphasizing the interpretation of structural semantics. Instance segmentation goes a step further by segmenting each individual object instance into separate regions and assigning a unique identifier, enabling the distinction between different instances of the same class. In medical image segmentation, the primary objective is to accurately identify and label anatomical structures, organs, or lesions as specific semantic categories. This process does not require differentiation between individual instances within the same category but rather focuses on determining overall category affiliation. Therefore, the task definition of medical image segmentation aligns closely with that of semantic segmentation, where the accurate delineation of anatomical structures and lesions is achieved through fine-grained pixel-level classification.

Among the existing medical image semantic segmentation methods, Fully Convolutional Network (FCN), SegNet, and U-Net are considered the most representative architectures. FCN achieves end-to-end semantic segmentation by directly producing dense pixel-level predictions through fully convolutional layers. In contrast, SegNet adopts an encoder–decoder architecture, where the decoder utilizes max-pooling indices stored during encoding to perform upsampling, thereby restoring spatial resolution. U-Net stands out due to its distinctive skip connection design, which fuses feature maps from corresponding encoder and decoder layers. This mechanism effectively combines global and local features, enabling the model to capture more detailed and nuanced representations, thus significantly improving segmentation accuracy. Shen Y. et al. [26] demonstrated the superiority of U-Net over FCN and SegNet for liver segmentation through comparative experiments. At present, U-Net has become the baseline model in liver segmentation, leading to the emergence of numerous improved variants. Rahman H. et al. [27] proposed ResU-Net for liver segmentation, which incorporates residual blocks into the U-Net framework. Compared with the traditional U-Net, ResU-Net enables more efficient feature extraction from input data. Ansari M.Y. et al. [28] introduced Dense-PSP-UNet, an extension of Dense-UNet that incorporates a modified Pyramid Scene Parsing (PSP) module into the skip connections to capture multi-scale features and contextual relationships.

Inspired by the dense connectivity of DenseNet and the limitations of simple skip connections in U-Net, Zhou Z. et al. [29] proposed U-Net++ for liver segmentation. U-Net++ introduces nested and dense skip connections to reduce the semantic gap between feature maps and improve the utilization of hierarchical features.

The emergence of attention mechanisms has encouraged their integration with U-Net architectures, owing to their alignment with biological visual processing. Attention mechanisms mimic the human visual system’s ability to focus on salient information in complex scenes. Li J. et al. [30] proposed Eres-UNet++ for liver segmentation, employing ResNet-34 as the encoder and integrating the U-Net++ design. Efficient Channel Attention modules (ECA) are embedded in each downsampling stage to enhance liver-specific feature extraction while suppressing irrelevant information. Wang J. et al. [31] developed EAR-U-Net for liver segmentation, using EfficientNetB4 as the encoder to extract richer features. Attention gating is applied in the skip connections to emphasize task-relevant features, and residual blocks replace decoder convolutions to enhance segmentation accuracy. Wang Z. et al. [32] introduced HDA-ResUNet, which replaces the bottleneck layer with hybrid dilated convolutions to capture multi-scale features and contextual information. Channel attention modules are incorporated into skip connections to enhance the extraction of relevant features. Wu J. et al. [33] proposed MSA-UNet, which enhances liver segmentation through several innovations. Multi-scale residual blocks (MSRBs) in the encoder improve feature extraction, while multi-scale attention modules (MSAMs) in the decoder strengthen relevant features and suppress noise. The bottleneck integrates an attention-guided atrous spatial pyramid pooling (AASPP) module to capture high-level semantics, and residual attention skip connections (RASM) are used to reduce semantic gaps between encoders and decoders.

The inability of local convolutions to model long-range dependencies limits the global context representation of CNN-based segmentation networks. As a result, researchers have begun combining U-Net with Transformer modules for liver semantic segmentation, with TransU-Net and Swin-Unet being the most prominent examples. Li L. et al. [34] developed RDCTransU-Net for liver segmentation, featuring a ResNeXt5-based encoder enhanced with dilated convolutions and a Transformer module at its tail to jointly capture local-global features. Wang B. et al. [35] developed multi-scale TransUNet++, which draws on the U-Net++ structure by reintroducing skip connections and incorporating a CNN–Transformer module at the bottleneck to improve global context modeling. Zhang J. et al. [36] introduced ST-Unet for liver segmentation, featuring an encoder built upon a CNN–Transformer hybrid architecture with Swin Transformer modules to enhance feature extraction. A novel cross-layer feature enhancement (CLFE) module enables effective inter-layer learning, while spatial and channel squeeze-and-excitation modules are employed to highlight salient regions.

To enhance model generalizability while safeguarding patient privacy, federated learning (FL) has emerged as a key research direction in medical image segmentation. FL enables collaborative training across multiple institutions without the need to share raw data, facilitating a balance between data privacy and model performance. Bernecker T. et al. [37] proposed the FedNorm and FedNorm+ algorithms, which address liver segmentation tasks by applying modality normalization strategies to effectively mitigate discrepancies between multimodal data such as CT and MRI. Fu H. et al. [38] developed PAF-Fed, a personalized FL framework that integrates partial parameter sharing, Fourier transformation, and self-attention mechanisms to enhance modeling capabilities for heterogeneous data in multi-organ abdominal segmentation. Hsiao C.H. et al. [39] introduced the Hybrid-ResUNet model, which combines 2D and 3D deep learning architectures and incorporates transfer learning and the FedAvg optimization strategy to achieve accurate liver and tumor segmentation while preserving privacy and improving generalization.

As the application of deep learning in medical imaging expands, model interpretability has gained increasing attention. To improve the transparency and trustworthiness of models in clinical decision making, many studies have introduced visualization techniques to enhance the interpretability of model outputs. Brunese M.C. et al. [40] and Halder A. et al. [41] integrated the Grad-CAM technique into U-Net and its variants to generate heatmaps that highlight model attention areas, aiding clinicians in interpreting liver structures. Anil B.C. et al. [42] proposed an explainable AI (XAI) approach combining cascaded CNNs with gray-level co-occurrence matrix (GLCM) texture features for liver cancer identification. This method enhances the traceability and reliability of diagnostic outcomes through feature visualization and importance analysis.

Despite significant advances in liver semantic segmentation techniques, the current methods still face notable limitations that hinder further performance improvements and clinical adoption. Traditional methods are computationally efficient and highly interpretable; however, they rely heavily on manually set parameters and lack generalizability, making them inadequate for capturing the complex anatomical variability of the liver. CNN-based approaches demonstrate strong local feature extraction capabilities, with relatively simple architectures and stable training. Nevertheless, their limited receptive field restricts global context modeling, impeding the capture of long-range dependencies and thus affecting the accuracy of comprehensive anatomical segmentation. Enhanced U-Net variants improve fine-grained representation through multi-scale feature fusion. However, cross-layer semantic integration remains suboptimal, with prevalent issues such as feature redundancy and semantic inconsistency. Attention-enhanced models improve focus on salient regions and suppress background noise, thereby enhancing segmentation accuracy. Yet, they still lack robust global perception, and the guidance provided by attention mechanisms can be prone to error. Hybrid CNN-Transformer architectures integrate both local feature extraction and global context modeling, offering high adaptability. However, their complex structures pose challenges in feature fusion and semantic consistency, which remain areas for improvement. Federated learning enables privacy-preserving collaborative training across institutions, enhancing model generalization and making it suitable for settings with stringent compliance requirements. However, high communication overhead and significant inter-institutional data heterogeneity can negatively impact model aggregation, limiting its practical deployment. Explainable AI (XAI) methods enhance model transparency and trustworthiness, which is crucial for clinical applications. Nonetheless, their effectiveness is often constrained by underlying model architectures, and the lack of standardized evaluation metrics poses challenges in meeting clinical demands for stability and reproducibility. The specific limitations are summarized in Table 2.

A thorough analysis of the current technologies reveals that mainstream liver semantic segmentation methods still exhibit significant research gaps across several critical dimensions, as outlined below: (1) The liver exhibits significant morphological heterogeneity in medical images, including variations in shape, size, texture, and pathological changes. These factors pose substantial challenges to the encoder’s feature extraction capacity. The existing methods struggle to capture such complexity, particularly due to inadequate multi-scale feature representation. (2) Due to the intrinsic limitations of convolutional operations, the current segmentation networks exhibit deficiencies in global feature learning. The local receptive fields of convolutional neural networks (CNNs) constrain their ability to capture holistic structural features of the liver, thereby reducing segmentation accuracy. (3) The commonly used skip connection architecture relies primarily on unidirectional information flow from an encoder to a decoder, disregarding the semantic discrepancies across different encoder layers. This unidirectional flow limits multi-level feature integration, resulting in insufficient fusion between encoder and decoder features, which may cause under-segmentation or over-segmentation. (4) The current deep learning-based liver segmentation models often function as black boxes, lacking transparency and interpretability in their decision-making processes. This limitation undermines clinical trust and hinders further model optimization.

To address these challenges, a novel network architecture is proposed for the semantic segmentation of liver CT images. The key contributions of the proposed method are summarized as follows: (1) A pyramid-structured multi-scale convolutional attention (MSCA) module is embedded at various stages of the encoder to automatically focus on critical regions in the image while capturing features at multiple scales. This enhances the encoder’s adaptability to complex liver morphologies and facilitates more effective feature aggregation and extraction. (2) Atrous spatial pyramid pooling (ASPP) is introduced in the bottleneck layer. By utilizing convolutions with varying dilation rates and incorporating global pooling, the receptive field is effectively expanded. This enhances the model’s ability to capture rich global contextual information, which is critical for understanding the spatial relationships between the liver and surrounding structures. (3) The original skip connections are replaced with a Channel Transformer module, comprising a Channel-wise Cross Fusion Transformer (CCT) and Channel-wise Cross-Attention (CCA). The CCT adopts a Transformer-based structure to overcome the CNN’s limitations in modeling long-range dependencies, improving global feature comprehension and enabling more effective inter-channel feature aggregation in the decoder. The CCA module further fuses features from the CCT with those from the decoder, narrowing the semantic gap between the encoder and decoder layers. (4) To enhance model transparency and interpretability, a comprehensive heatmap-based visualization analysis was conducted on the proposed architecture, covering the encoder, bottleneck, and decoder components. This visual interpretability offers valuable insights into the feature extraction and fusion processes, facilitating further model refinement.

## 2. Materials and Methods

### 2.1. Original U-Net

**Figure 1:**
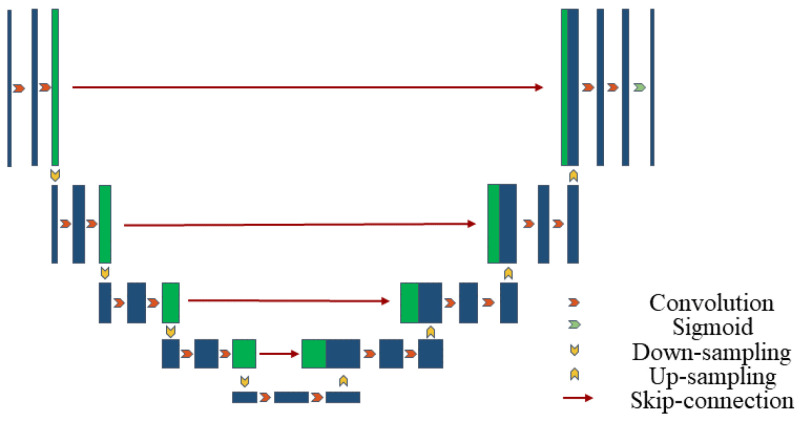
U-Net architecture.

U-Net is a convolutional neural network structure for image segmentation, consisting of an encoder for feature extraction and a decoder for generating segmentation results [43]. Below is the mathematical representation of the basic U-Net structure in Figure 1.

Let the input image be X and the output segmentation result be $\hat{Y}$. U-Net consists of an encoder (downsampling path), a bottleneck layer and a decoder (upsampling path). The entire network encoding process is shown in Equation (1):

(1) Xencodern/2=Conv(Hencodern/2−1,Wencodern/2)Hencodern/2=ReLU(Xencodern/2)

where Conv(X,W) represents the convolution operation, W is the convolution kernel, and ReLU(X) represents the rectified linear unit activation function. This includes the encoder layer and the bottleneck layer.

The entire network decoding process is shown in Equation (2):

(2) Xdecodern−1=ConvTranspose(Hdecodern/2+2,Wdecodern−1)Hdecodern−1=ReLU(Xdecodern−1)Yn=Conv(Hdecodern−1,Wdecodern)Y^=Sigmoid(Yn)

where ConvTranspose(X,W) represents the transposed convolution operation, Sigmoid(X) represents the Sigmoid activation function, and $\hat{Y}$ is the network output.

In each layer of the decoder, the feature maps from the corresponding layer of the encoder are concatenated, as shown in Equation (3):

(3) $${\hat{H}}_{i}=Concatenate(H_{i},X_{n-i})$$

The output of the entire U-Net network can be expressed as shown in Equation (4):

(4) $$\hat{Y}=Sigmoid(Conv(Concatenate(H_{encoder_{n}/2},X_{decoder_{n}/2+1}),W_{n+1}))$$

This is a simplified representation. The actual U-Net structure may include more details and layers. However, this representation captures the main architecture of U-Net, including the encoder, bottleneck layer, decoder, and skip connections.

### 2.2. Improved Feature Extraction Module

In the domain of liver semantic segmentation, the encoder of the U-Net architecture serves as the core component for feature extraction, playing a decisive role in accurately capturing the multi-level and detailed features inherent in liver imaging. Recent studies have focused on integrating attention mechanisms into the U-Net encoder, aiming to dynamically assign differentiated weights to various elements of the input data. This strategy enables the network to selectively focus on feature regions that are critical to the segmentation task [44]. Such innovation not only enhances the model’s sensitivity to essential features but also effectively mitigates the influence of irrelevant features, thereby significantly improving both the efficiency and accuracy of feature extraction.

Among the various implementations of attention mechanisms, channel attention [45] and spatial attention [46] have received particular attention. The channel attention mechanism assesses the importance of each channel within the feature map, allowing the model to automatically assign higher weights to the more relevant channels. This process reinforces the representation of important features while suppressing noise from less critical ones. Conversely, the spatial attention mechanism operates on the spatial dimensions of the feature map, amplifying region-specific features that are closely associated with liver segmentation, thereby facilitating more precise localization and delineation of target structures.

However, due to the inherent complexity of liver anatomy and significant inter-patient variability—especially with respect to scale variations and the presence of small or discontinuous targets—the existing methods still encounter limitations in effective feature extraction. To address this challenge and further improve the model’s capability in extracting relevant liver features, this study draws inspiration from [47] and introduces a multi-scale convolutional attention (MSCA) mechanism.

**Figure 2:**
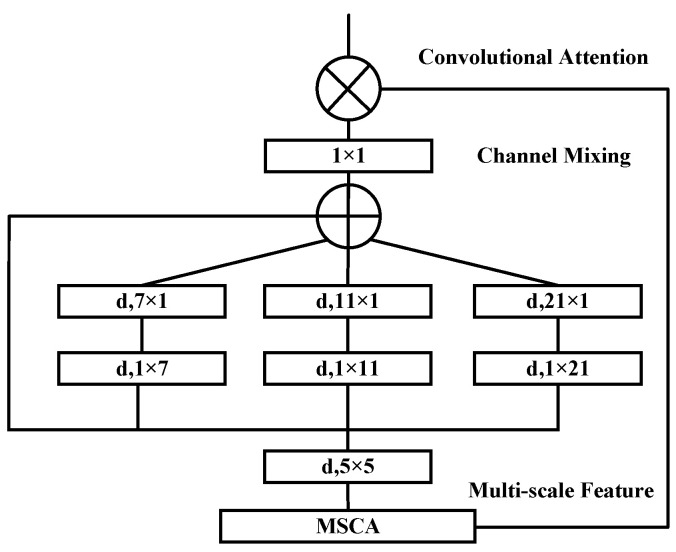
Structure diagram of multi-scale convolutional attention (MSCA).

The MSCA mechanism adopts a pyramid-like structure and comprises three key components: depth-wise convolutions for local information extraction; multi-branch separable depth-wise convolutions to capture features at multiple scales; and a 1 × 1 convolution to model inter-channel relationships. The output of the 1 × 1 convolution serves as the attention weight, which is used to reweight the input features, thereby producing the refined output of the MSCA module. The detailed structure is illustrated in Figure 2.

From a mathematical expression perspective, this process can be formulated as shown in Equation (5):

(5) $$\begin{array}{l} input=F\in R^{C\times H\times W}\\ A_{0}=DW-Conv_{5\times 5}(F)\\ A_{1}\;=SDW-Conv_{\left(7\times 1;1\times 7\right)}(A_{0}\;)\\ A_{2}=SDW-Conv_{\left(11\times 1;1\times 11\right)}(A_{0})\\ A_{3}\;=SDW-Conv_{\left(21\times 1;1\times 21\right)}(A_{0})\\ A=A_{0}\;+A_{1}\;+A_{2}\;+A_{3}\\ A_{out}\;=Conv_{1\times 1}(A)\\ Attention=A_{out\;}\\ Out=Attention⊗F \end{array}$$

where F represents the input features, Attention and Out represent the attention mapping and output, respectively, ⊗ represents element-wise matrix multiplication operation, DW-Conv represents depth-wise convolution, SDW-Conv represents separable depth-wise convolution, and $Conv_{1\times 1}$ represents 1 × 1 convolution. In the design of multi-scale convolutional attention, the three branches have kernel sizes of 7, 11, and 21, mimicking standard depth-wise convolutions with large kernels. By decomposing a given k×k depth-wise convolution kernel into 1×k and k×1 separable depth-wise convolution kernels, it can simulate features at different scales through branches with different kernel sizes, enriching feature representation. Separable depth-wise convolution, due to its lightweight nature, requires only one set of 1×k and k×1 convolutions to approximate a standard 2D convolution with a k×k kernel, effectively reducing computational overhead.

### 2.3. Improved Bottleneck Layer Module

The bottleneck layer of the U-Net architecture serves as the core of the network, not only bridging the encoder and decoder but also bearing the critical responsibility of feature compression and integration. As the encoding process progresses, the spatial resolution of the image gradually decreases, while the level of feature abstraction correspondingly increases. This hierarchical encoding enables the network to effectively capture global structural information. By the time the data flow reaches the bottleneck layer, the network has distilled a set of highly condensed and semantically rich feature representations. Although these features exist at a lower spatial resolution, they carry abundant contextual information. However, the inherent limitation of the convolutional receptive field constrains the model’s ability to fully capture global dependencies.

To further enhance the performance of liver semantic segmentation—particularly by strengthening the expressive power of features at the bottleneck layer—dilated convolutions have emerged as an effective solution [48]. The unique advantage of dilated convolutions lies in their ability to significantly expand the receptive field of the convolutional kernel without sacrificing spatial resolution. This property enables the model to access broader contextual information while preserving spatial relationships between pixels. More importantly, stacking dilated convolution layers with varying dilation rates allows the model to naturally incorporate multi-scale information. This capability is particularly beneficial in complex medical image segmentation tasks, where distinguishing anatomical structures of varying sizes is essential for accurate interpretation.

**Figure 3:**
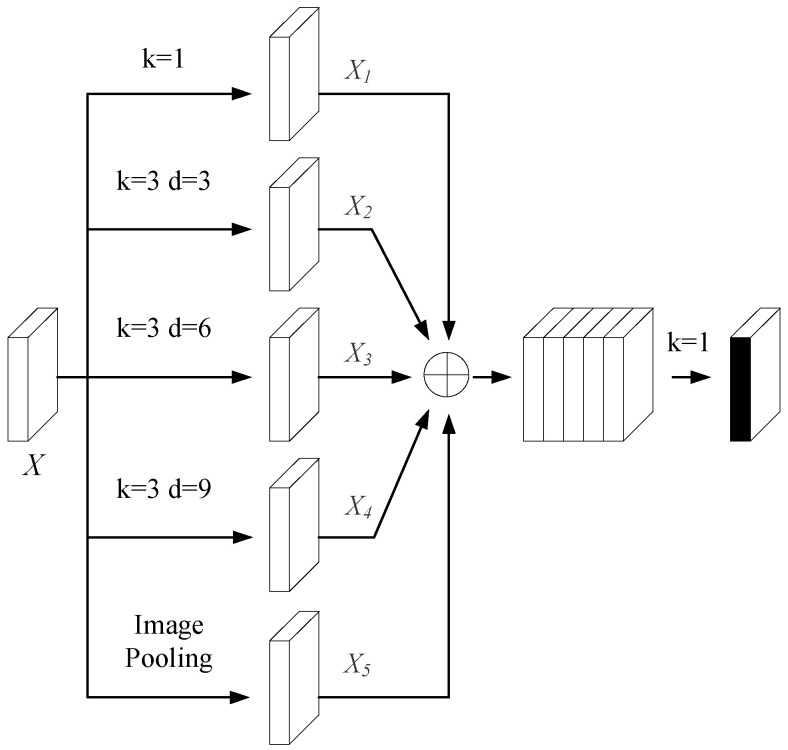
Structure of the ASPP module.

Given the bottleneck layer’s pivotal role in semantic feature extraction, this study incorporates the atrous spatial pyramid pooling (ASPP) module from DeepLabv3+ [49] to further enhance its capacity for global context modeling. The ASPP module employs dilated convolutions with different dilation rates to construct a pyramid-like architecture that captures features at multiple scales. As shown in Figure 3, the ASPP module consists of a 1 × 1 convolution, three parallel 3 × 3 convolutions with distinct dilation rates, and a global average pooling branch. The outputs of all the branches are concatenated and followed by a batch normalization operation, enabling the efficient fusion of features across varying receptive fields.

The output feature map of the dilated convolution is defined as shown in Equation (6):

(6) $$y(i,j)=\sum_{u=0}^{H}\sum_{v=0}^{W}x(i+ar\times u,j+ar\times v)\times w(u,v)$$

where H,W represents the length and width of input features; x(i,j) represents the pixel values of (i,j) in the image; ar represents the dilation rate; y(i,j) represents the output of the input features after dilated convolution; (u,v) represents the coordinates of the convolution kernel center (0≤u≤H,0≤v≤W); and w(u,v) represents the weight corresponding to the convolution kernel coordinate.

The output of the global average pooling layer is defined as shown in Equation (7):

(7) $$X_{5}^{L}(c,1,1)=\frac{1}{H\times W}\sum_{i=1}^{H}\sum_{j=1}^{W}X(c,i,j)$$

where X(c,i,j) represents the pixel value of the coordinate in the c channel of the input feature map X; $X_{5}^{L}(c,1,1)$ represents the output feature map.

In the ASPP module, five branches provide five differently sized receptive fields. Convolution layers with larger dilation rates capture more comprehensive global contextual features, while those with smaller rates add detailed features to the deep network. Inserting the ASPP module in the transition layer acquires multi-scale features of varying receptive fields. The first four convolution operations alter only the channel dimension, yielding $X_{1}$, $X_{2}$, $X_{3}$, and $X_{4}$. The feature map $X_{5}^{L}$ obtained after global average pooling is upsampled via bilinear interpolation to restore its spatial dimensions to those of the input feature map, thereby yielding the refined $X_{5}$. Finally, all the processed feature maps are concatenated along the channel dimension, with the specific result shown in Equation (8):

(8) $$\begin{array}{l} X(c,H,W){|}_{0\leq c\leq 5C}=Concatenate(X_{1}(c,H,W){|}_{0\leq c\leq C},X_{2}(c,H,W){|}_{0\leq c\leq C},\\ X_{3}(c,H,W){|}_{0\leq c\leq C},X_{4}(c,H,W){|}_{0\leq c\leq C},X_{5}(c,H,W){|}_{0\leq c\leq C} \end{array}$$

where C,H,W represent the channel number, length, and width of a feature map, respectively. After concatenation, a 1 × 1 convolution is applied. This not only compresses the feature map to a specified dimension but also, in the channel dimension, realizes the interaction of multi-scale information in the bottleneck layer feature map. Thus, it lays the foundation for image reconstruction in the decoding layer.

### 2.4. Improved Feature Fusion Module

The introduction of skip connections in U-Net was originally motivated by the need to mitigate the issue of feature loss commonly observed in deep convolutional neural networks during information propagation. By directly connecting shallow features from the encoder with corresponding deeper features in the decoder, skip connections not only facilitate inter-layer feature transmission but also enhance the network’s ability to preserve fine-grained image details. Although skip connections have demonstrated remarkable effectiveness within the U-Net framework, recent studies have identified their limitations in addressing the semantic gap problem [50,51]. The semantic gap refers to the discrepancy in semantic representation between shallow and deep features; directly fusing such features may lead to semantic misalignment, thereby impairing segmentation accuracy.

To address this challenge, various strategies have been proposed. These include leveraging attention mechanisms to dynamically reweight features from different layers for more refined fusion [31,32], incorporating residual or dense learning paradigms to strengthen feature propagation and reuse [29,30], and employing multi-scale feature fusion methods to enrich representation and improve adaptability to targets of varying sizes [28]. While these approaches partially alleviate the semantic gap, they do not fundamentally resolve its underlying cause. Furthermore, the locality of convolution operations inherently limits the global modeling capability of U-Net [52,53].

In light of these issues, and inspired by the work in [50], this study proposes replacing the conventional skip connection mechanism with a channel-wise Transformer module to further improve the performance of U-Net in medical image segmentation—particularly in addressing the semantic gap and enhancing global context modeling. The proposed module consists of two components: the Channel-wise Cross Fusion Transformer (CCT) and the Channel-wise Cross-Attention (CCA) module. The CCT module is designed to strengthen the relationships among multi-scale features extracted from the encoder and comprises three submodules: multi-scale feature embedding, multi-channel cross-attention, and a multi-layer perceptron (MLP).

In the multi-scale feature embedding process, firstly the outputs $E_{i}\in R^{\frac{HW}{i^{2}}\times C_{i}}(i=1,2,3,4)$ of the 4 skip connection layers are given. The features of four different sizes are reshaped and flattened in 2D with sizes of P,P/2,P/4,P/8 respectively. The feature reshaping aims to map each feature to the same region of the encoder features at four scales while maintaining the original channel size. Then, the tokens $T_{i}(i=1,2,3,4)\in R^{\frac{HW}{i^{2}}\times C_{i}}$ of the four layers are concatenated, where $T_{i}(i=1,2,3,4)$ represents the key and $T_{\sum }=Concat(T_{1},T_{2},T_{3},T_{4})$ represents the value.

In the multi-head cross-attention mechanism, Tokens are fed into the multi-head cross-attention module, and then through a residual-structured MLP to encode channel relationships and dependencies and to refine features from different U-Net encoder levels using multi-scale features.

**Figure 4:**
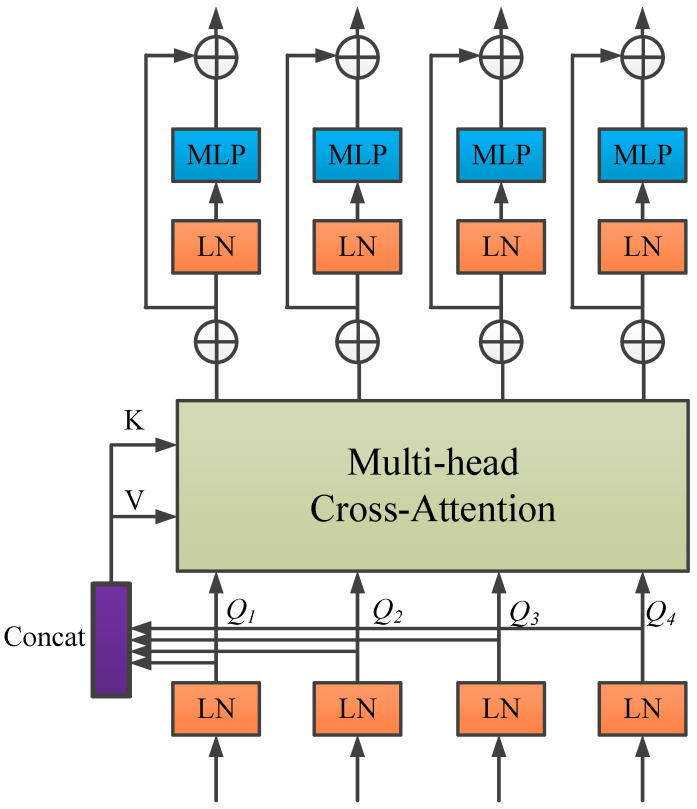
CCT structure.

As shown in Figure 4, the CCT module has five inputs: four tokens $T_{i}$ as queries, one cascaded token $T_{\sum }$ as the key, and value. The mathematical formulation is explicitly given in Equation (9):

(9) $$Q_{i}=T_{i}W_{Q_{i}},K=T_{\sum }W_{K},V=T_{\sum }W_{V}$$

where $W_{Q_{i}}\in R^{C_{i}\times d},W_{K}\in R^{C_{\sum }\times d},W_{V}\in R^{C_{\sum }\times d}$ represents weights under different inputs, d is the sequence length, and $C_{i}(i=1,2,3,4)$ denotes the channel dimensions of the four skip connection layers. Here, the channel dimensions should match the feature map dimensions, i.e., $C_{1}=64,C_{2}=128,C_{3}=256,C_{4}=512$.

When $Q_{i}\in R^{C_{i}\times d},K\in R^{C_{\sum }\times d},V\in R^{C_{\sum }\times d}$ are obtained, a similarity matrix $M_{i}$ is generated via the cross-attention (*CA*) mechanism, and *V* is weighted by $M_{i}$. This can be mathematically expressed by Equation (10):

(10) $$CA_{i}=M_{i}V^{T}=\sigma \left[\psi \left(\frac{{Q_{i}}^{T}K}{\sqrt{C_{\Sigma }}}\right)\right]V^{T}=\sigma \left[\psi \left(\frac{{W_{Q_{i}}}^{T}{T_{i}}^{T}T_{\Sigma }W_{K}}{\sqrt{C_{\Sigma }}}\right)\right]{W_{V}}^{T}{T_{\Sigma }}^{T}$$

where σ(⋅) and ψ(⋅) represent instance normalization and softmax function, respectively.

Under the N head attention, the output calculation formula after multi-head cross-attention is shown in Equation (11):

(11) $$MCA_{i}=\frac{1}{N}\sum_{j=1}^{N}CA_{i}^{j}$$

where N stands for the number of heads. Applying MLP and residual operators, the final output is shown in Equation (12):

(12) $$Q_{i}=MCA_{i}+MLP(Q_{i}+MCA_{i})$$

For simplicity, instance normalization (LN) in the equations is omitted here. In the above equation, repeating operations L times represents constructing a Transformer with L layers. As described in the literature [50], setting L and N to 4 achieves optimal performance. Finally, the four outputs of the L layer $O_{1},O_{2},O_{3},O_{4}$ are reconstructed via upsampling and convolutional layers, and then connected to decoder features $D_{1},D_{2},D_{3},D_{4}$, respectively.

**Figure 5:**
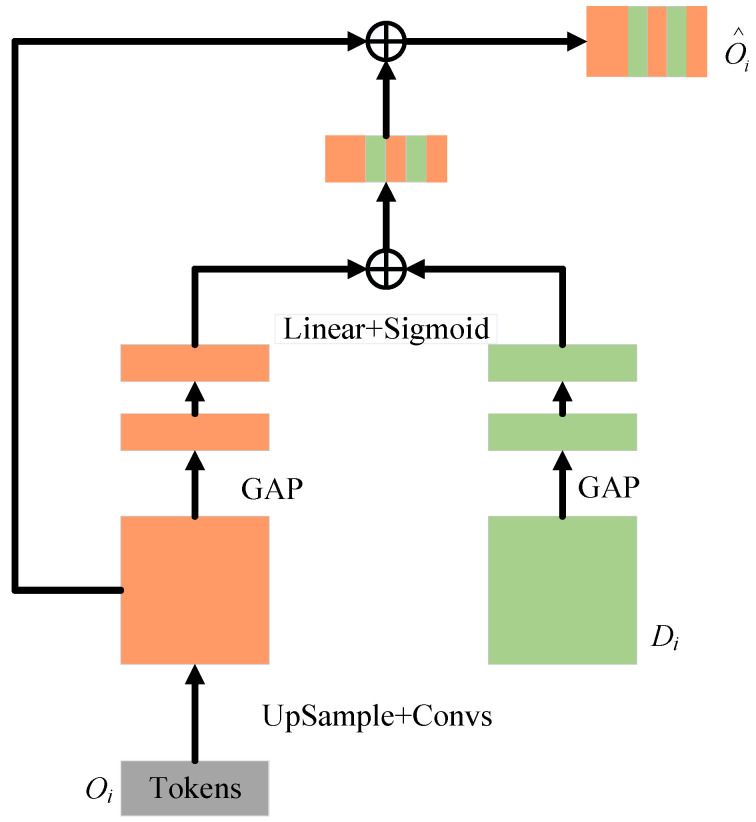
CCA structure.

To better fuse semantically inconsistent features between the Channel Transformer and U-Net decoder, the CCA module in Figure 5 is used. It can guide the channel and information filtering of Transformer features, eliminating ambiguities with decoder features.

From a mathematical perspective, the output $O_{i}\in R^{C\times H\times W}$ of i Channel Transformer and the feature map $D_{i}\in R^{C\times H\times W}$ of i decoder are used as inputs for the cross-attention mechanism. Spatial compression is implemented via global average pooling, resulting in vector $\eta (X)\in R^{C\times 1\times 1}$, with $\eta (X)=\frac{1}{H\times W}\sum_{i=1}^{H}\sum_{j=1}^{W}X^{k}(i,j)$ of its k channel. These operations are used to embed global spatial information and ultimately generate the attention mask, and these operations are formally expressed by Equation (13):

(13) $$M_{i}=L_{1}\eta (O_{i})+L_{2}\eta (D_{i})$$

where $L_{1}\in R^{C\times C}$ and $L_{2}\in R^{C\times C}$ represent the weights of two Linear layers and the ReLU operator δ(⋅). The formula encodes channel dependencies. As indicated by ECA-Net, avoiding dimension reduction is crucial for learning channel attention [50]. A single Linear layer and sigmoid function construct the channel attention map. The resulting vector recalibrates or activates $O_{i}$ to $\overset{\wedge }{O_{i}}=\sigma (M_{i})\cdot O_{i}$, where $\sigma (M_{i})$ denotes channel importance. Finally, the masked $\overset{\wedge }{O_{i}}$ is concatenated with the upsampled features of the i decoder level.

The CCT module is designed to construct multi-scale contextual information based on multi-channel encoder features, thereby enhancing the model’s global modeling capability. It effectively alleviates semantic discrepancies among different encoder channels, strengthens inter-channel correlations, and enables cross-channel feature fusion. On the other hand, the CCA module aims to reduce the semantic gap between the encoder and decoder. By integrating the CCA-processed features with the deep features from the bottleneck layer, it facilitates the effective fusion of high-level semantic information and low-level spatial details. Collectively, the synergy between the CCT and CCA modules contributes to significantly bridging the semantic gap between the encoder and decoder, thereby enhancing the overall representational capacity of the model.

### 2.5. Multi-Channel Key Feature Deep Extraction and Cross Fusion Network Architecture

**Figure 6:**
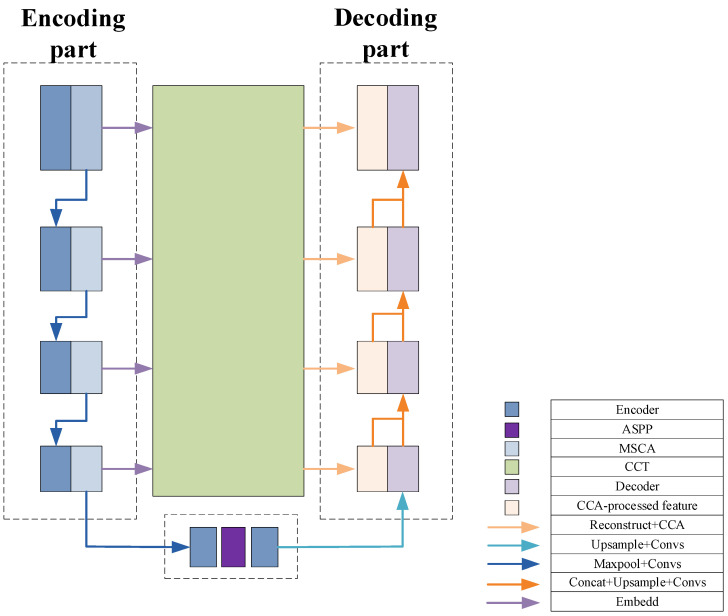
Improved network architecture.

To better understand the feature propagation process of the proposed network structure, namely TAMU-Net (Transformer-ASPP-MSCAU-Net), this section provides a summary of the network architecture, as shown in Figure 6.

In the feature processing pipeline, the encoder first applies the MSCA module to refine the input features. This strategy specifically addresses the common scale variation issue in liver slices and effectively extracts and strengthens multi-level feature information.

The design of the MSCA module balances efficiency and representational power, with its main innovation lying in the integration and enhancement of conventional channel and spatial attention mechanisms [45,46]. Unlike traditional channel attention methods that rely solely on global average pooling to generate channel weights, MSCA incorporates parallel branches with convolutional kernels of varying sizes, thereby preserving inter-channel dependency modeling while enhancing responsiveness to multi-scale features. Furthermore, MSCA employs a learnable fusion mechanism to adaptively regulate the contributions of each branch, improving the flexibility and precision of channel weighting.

Meanwhile, compared to spatial attention mechanisms that first compress channels before computing spatial attention maps, MSCA introduces multi-scale convolutions directly within the channel modeling process. This approach effectively preserves spatial structural features, prevents the degradation of spatial representation, and reduces module redundancy by unifying feature modeling in a single pathway, thereby enhancing computational efficiency.

Overall, MSCA integrates scale-aware responses with channel modeling in a compact architecture, overcoming the limitations of separated channel and spatial attention. This results in superior structural adaptability and representational capacity.

These features are then passed to the bottleneck layer. At this stage, by incorporating the ASPP architecture, dilated convolutions with different expansion rates capture a diversified receptive field ranging from local to global. Global average pooling is used to aggregate features, significantly enhancing the ability to integrate global contextual information.

To further optimize feature representation and reduce the semantic gap between deep and shallow features, this study employs the Channel Transformer module to replace traditional skip connections. This mechanism not only facilitates direct communication between features at different levels within the encoder, but also enhances the deep fusion of abstract deep features with detailed shallow features, ensuring coherence and completeness of the information.

The Channel Transformer module balances architectural coherence and expressive flexibility in its fusion design, with its innovation centered on the deep interaction and adaptive integration of cross-level, multi-scale semantic information. Unlike MCTrans [54], which relies on a single-path Transformer for multi-scale semantic modeling, and MISSFormer [55], which explicitly separates semantic sharing from spatial alignment, the Channel Transformer achieves a unified fusion of semantic and structural information through multi-path feature propagation and fine-grained channel modeling. This enhances the synergistic representation of global and local features. Moreover, the fusion is consolidated within the decoding stage, preventing information fragmentation and path redundancy, thereby improving fusion efficiency and feature integration accuracy.

Overall, the Channel Transformer surpasses traditional Transformer-based skip connection fusion limitations, demonstrating superior performance in fusion pathway design, information integrity, and model adaptability.

Finally, the global features enhanced by the ASPP architecture are passed to the decoder. During decoding, these features are finely fused with the context-rich features passed through the Channel Transformer module. Through layer-wise upsampling and feature fusion operations, the spatial resolution of the image is gradually restored, and the segmentation boundaries are refined. This process ensures that the final output segmentation result retains global consistency while maintaining high detail accuracy, thereby achieving the efficient and precise segmentation of liver slices.

The specific mathematical expressions are given in Equation (14):

(14) $$\begin{array}{l} input=F\in R^{C\times H\times W}\\ F_{E_{n}}=MSCA(ConvF_{E_{n-1}})\\ F_{E_{5}}=ASPP(ConvF_{E_{4}})\\ F_{D_{m}}=Concatenate(CCA(CCTF_{E_{n}}),ConvF_{D_{m+1}})\\ output=Sigmoid(F_{D_{1}}) \end{array}$$

where MSCA is the multi-scale convolutional attention mechanism; ASPP is the atrous spatial pyramid pooling; CCA is the Channel-wise Cross-Attention module; and CCT is the module for Channel-wise Cross Fusion Transformer. $E_{n}$ represents the encoder layer and the bottleneck layer, and $D_{m}$ represents the decoder layer.

## 3. Segmentation Metrics

To comprehensively evaluate the model’s performance in 2D liver semantic segmentation tasks, this study selected six evaluation metrics, including Precision, Recall, Dice similarity coefficient (Dice), Intersection over Union (IoU), Mean Intersection over Union (MIoU), Mean Pixel Accuracy (MPA), and Accuracy, to assess the model’s performance in pixel-level classification tasks. These metrics are pixel-level measures, where higher values indicate better segmentation performance. True Positive (TP) refers to the number of pixels correctly predicted as liver, i.e., pixels that are actually liver and correctly classified as liver. False Negative (FN) refers to the number of liver pixels incorrectly predicted as background, i.e., liver pixels that are misclassified as background. True Negative (TN) refers to the number of pixels correctly predicted as background, i.e., background pixels that are correctly classified as background. False Positive (FP) refers to the number of background pixels incorrectly predicted as liver, i.e., background pixels that are misclassified as liver.

Precision: Precision is defined as the proportion of pixels actually belonging to the liver among all the pixels predicted as the liver, reflecting the exactness of the prediction outcomes. Precision is defined by Formula (15):

(15) $$P=\frac{TP}{TP+FP}$$

Recall: Recall is defined as the proportion of pixels correctly predicted as the liver relative to all the actual liver pixels, reflecting the sensitivity of the model. Recall is defined by Formula (16):

(16) $$R=\frac{TP}{TP+FN}$$

Dice similarity coefficient (Dice): Dice is a measure of the overlap between the predicted segmentation and the ground truth, emphasizing the harmonic mean of Precision and Recall. It quantifies the similarity between two sets by calculating twice the area of their intersection divided by the sum of their sizes. Dice is defined by Formula (17):

(17) $$Dice=\frac{2\times TP}{2\times TP+FP+FN}$$

Intersection over Union (IoU): Similar to Dice, IoU is also used to quantify the overlap between the predicted seg-mentation and the ground truth. IoU is defined as the ratio of the intersection area to the union area between the predicted segmentation and the ground truth. Compared to Dice, IoU is a more stringent metric. Therefore, the IoU value is generally lower than the Dice score. IoU is defined by Formula (18):

(18) $$IoU=\frac{TP}{FN+FP+TP}$$

Mean Intersection over Union (MIoU): MIoU is calculated by computing the IoU for each class individually and then taking the mean value across all the classes, providing an overall measure of segmentation performance. MIoU is defined by Formula (19):

(19) $$MIoU=\frac{1}{K+1}\sum_{i=0}^{k}\frac{TP}{FN+FP+TP}$$

where *K* + 1 denotes a total of *K* + 1 classes.

Mean Pixel Accuracy (MPA): MPA is derived by first calculating the proportion of correctly classified pixels for each class and then averaging these values across all the classes. MPA is defined by Formula (20):

(20) $$MPA=\frac{\sum_{i=0}^{k}p_{ii}}{(K+1)\sum_{i=0}^{k}\sum_{j=0}^{k}p_{ij}}$$

where K+1 denotes a total of K+1 classes; i represents the ground truth; j indicates the predicted value; $p_{ij}$ signifies the number of pixels that actually belong to class i but are predicted as class j; and $p_{ii}$ indicates the number of pixels that are correctly predicted.

Accuracy: Accuracy is a quantitative measure defined as the ratio of correctly classified pixels to the total number of pixels in the dataset, reflecting the model’s segmentation capability in distinguishing the liver and background regions. Accuracy is defined by Formula (21):

(21) $$Accuary=\frac{TP+TN}{TP+TN+FP+FN}$$

To fully leverage the spatial structural information along the Z-axis in CT volumetric data, we introduced five additional evaluation metrics for assessing the 3D reconstruction-based liver volume segmentation results: Dice similarity coefficient (DICE), Intersection over Union (IOU), Volume Overlap Error (VOE), Relative Volume Difference (RVD), Average Symmetric Surface Distance (ASD), and Maximum Symmetric Surface Distance (MSD). These metrics collectively enable a comprehensive evaluation of the model’s 3D segmentation performance from two critical dimensions: volumetric accuracy and boundary consistency. 

Dice similarity coefficient (DICE): DICE is defined as twice the volume of the intersection between the predicted segmentation and the ground truth divided by the sum of their volumes. DICE is defined by Formula (22):

(22) $$DICE=\frac{2\left|C_{pr}\cap C_{gr}\right|}{\left|C_{pr}\right|+\left|C_{gr}\right|}$$

where $C_{pr}$ represents the volume of the predicted sample, and $C_{gr}$ represents the volume of the ground truth sample.

Intersection over Union (IOU): IOU is defined as the ratio of the volume of the in-tersection to the volume of the union between the predicted segmentation and the ground truth. IOU is defined by Formula (23):

(23) $$IOU=\left|\frac{C_{pr}\cap C_{gr}}{C_{pr}\cup C_{gr}}\right|$$

It should be noted that Dice and IoU are commonly used as overlap evaluation metrics in 2D image segmentation tasks, while their uppercase forms, DICE and IOU, are typically used in 3D medical image segmentation to assess similarity at the voxel level.Volume Overlap Error (VOE): VOE, representing the error rate, is analogous to the DICE definition but uses subtraction instead of the AND operation, making it more sensitive in medical applications. A smaller VOE indicates a lower overlap error. VOE is defined by Formula (24):

(24) $$VOE=\frac{2\left|C_{pr}-C_{gr}\right|}{\left|C_{pr}\right|+\left|C_{gr}\right|}$$

Relative Volume Difference (RVD): RVD measures the volumetric difference between the predicted and ground truth results. A lower RVD suggests better segmentation performance. RVD is defined by Formula (25):

(25) $$RVD=(C_{pr}-C_{gr})/C_{gr}\times 100\%$$

Average Symmetric Surface Distance (ASD): ASD is the average distance between the surfaces of the predicted and ground truth results. A smaller distance implies better network performance. ASD is defined by Formula (26):

(26) $$ASD(A,B)=(\sum_{s_{A}\in S(A)}d(s_{A}\in S(B))+\sum_{s_{B}\in S(B)}d(s_{B}\in S(A)))/(\left|S(A)\right|+\left|S(B)\right|)$$

where S(A) represents the surface voxels in set A, and d(v,S(A)) denotes the shortest distance from any voxel to set S(A).

Maximum Symmetric Surface Distance (MSD): MSD evaluates the maximum symmetric distance between the predicted and ground truth results. A smaller MSD value indicates a higher matching degree between the two samples. MSD is defined by Formula (27):

(27) $$MSD(A,B)=\max(\underset{s_{A}\in S(A)}{\max}(d(s_{A}\in S(B))),\underset{s_{B}\in S(B)}{\max}(d(s_{B}\in S(A))))$$

## 4. Dataset and Experimental Setup

There are four main public datasets for liver and liver tumor segmentation: the LiTS (Liver Tumor Segmentation Challenge 2017) dataset, the SLIVER07 (Segmentation of the Liver Competition 2007) dataset, the 3D-IRCADb01 dataset, and the CHAOS (Combined CT-MR Healthy Abdominal Organ Segmentation) dataset.

The focus of this paper is on liver-based semantic segmentation, so only liver data needs to be extracted. The approach chosen for this study is supervised learning. Like other medical image datasets, the test sets typically do not have publicly available ground truth, so only liver data with ground truth annotations are selected.

Among the aforementioned datasets, LiTS contains 131 CT scans with ground truth annotations and is, therefore, included as experimental data. SLIVER07 provides 20 annotated CT scans and is also selected as experimental data. Since 3D-IRCADb01 is a subset of LiTS, it is excluded to avoid data redundancy. The CHAOS dataset includes both CT and MR modalities; to ensure consistency in data distribution, 20 CT scans with ground truth annotations are selected, and thus the CT subset of the CHAOS dataset is also included as experimental data.

**Figure 7:**
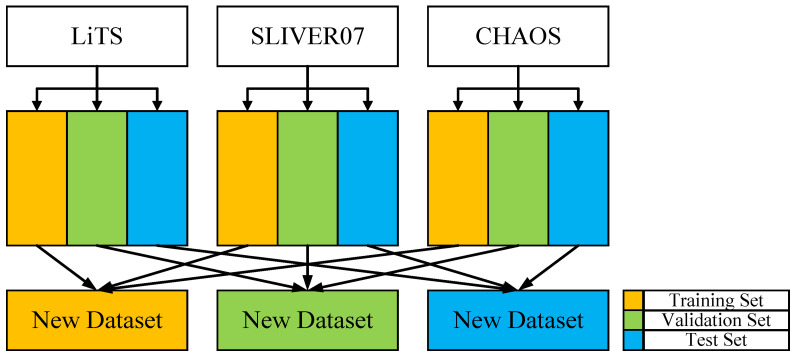
Dataset partitioning framework.

To prevent potential data leakage and ensure that slices from the same case do not appear in different data subsets, this study adopts a case-wise data splitting strategy to construct the training, validation, and test sets, thereby ensuring the independence and reliability of model evaluation. Additionally, to facilitate the comparison of 3D segmentation metrics, the Z-axis information of the CT images is preserved during dataset splitting. The final dataset is created by merging three different publicly available datasets. To maximize the utility of the three datasets (LiTS, SLIVER07, and CHAOS), each dataset is split into training, testing, and validation sets at a ratio of 8:1:1, ensuring no overlap between the sets. The three publicly available datasets are merged by categorizing liver data according to segmentation labels, mixing liver data from the training sets of each dataset, with the test and validation sets processed similarly. To reduce interference from background regions, slices without liver areas were excluded from the training set, thereby increasing the proportion of effective samples. The dataset partitioning framework and the corresponding quantitative results are presented in Figure 7 and Table 3, respectively.

This study integrates imaging and annotation data from three publicly available liver CT datasets—LiTS, SLIVER07, and CHAOS—to construct a larger, more finely annotated, and more diverse training and testing dataset. Specifically, the LiTS dataset contains a substantial number of CT scans with liver lesions, providing rich pathological features that facilitate the model’s learning of diseased liver characteristics. In contrast, the SLIVER07 and CHAOS datasets offer high-resolution, high-quality liver CT scans with expert annotations, which enhance the model’s ability to recognize normal liver structures.

The combination of these datasets not only significantly increases the volume and diversity of training samples but also improves the model’s adaptability to various types of CT imaging data.

**Figure 8:**
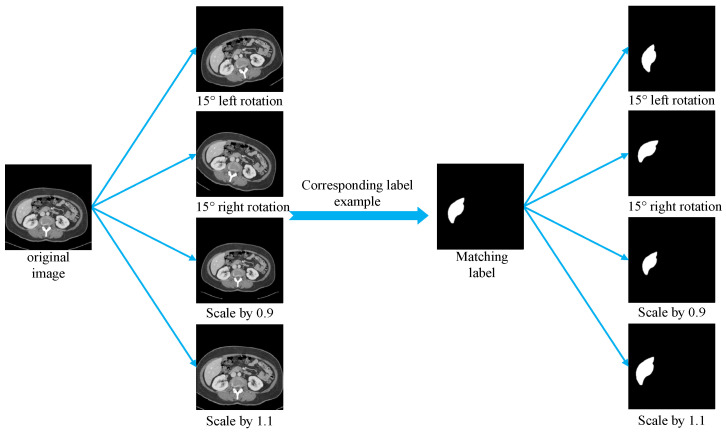
Examples of data augmentation.

This section establishes a standardized preprocessing pipeline for the LiTS, SLIVER07, and CHAOS liver CT datasets to enhance training consistency across heterogeneous sources and improve model generalization. Due to significant variations in scanning parameters and voxel sizes among datasets, all the images were resampled to a uniform spatial resolution. Although liver tissue typically falls within a Hounsfield Unit (HU) range of [40, 70], the CT values were clipped to the broader interval of [−400, 400] to eliminate irrelevant structures. The clipped HU values were then linearly normalized and mapped to the [0, 255] range in the uint8 format to facilitate compatibility with neural network input requirements. All the 2D slices were resized to 256 × 256 pixels using bilinear interpolation to meet the model input specifications while preserving anatomical structures and image detail. The preprocessed images were saved in the JPG format, and the corresponding annotations in the PNG format, ensuring consistency and efficiency in data loading during training. To improve model robustness and mitigate overfitting caused by the limited training data, several data augmentation techniques were applied during training, including random rotations (±15°) and random scaling (scale factor range: 0.9 to 1.1). These augmentations increased sample diversity while preserving the essential structural information of the original images, thereby enhancing the model’s ability to handle spatial transformations, scale variations, and anatomical heterogeneity, which ultimately contributes to improved generalization performance. Figure 8 presents comparative examples of liver CT images before and after augmentation.

In this experiment, data augmentation was applied only during the training phase, while the validation and test sets remained in their original states without any form of augmentation, ensuring the objectivity and comparability of the model evaluation. The specific data partition is as follows: prior to augmentation, the training set contained 18,228 image slices, while the validation and test sets included 4893 and 12,452 slices, respectively.

During training, the images in the training set were randomly augmented in real time upon each loading, including rotations (±15°) and scaling (scale range 0.9–1.1). Since the augmentation was dynamically applied at the image loading stage, it was inherently random and non-deterministic. Consequently, the same image slice could exhibit different augmented variants in each training epoch.

Taking 50 training epochs as an example, each slice could potentially present up to 50 different augmented versions. This strategy significantly expands the distribution and morphological diversity of the training data, thereby enhancing the model’s adaptability to variations in liver shape, scale, and spatial structure, and improving its robustness and generalization capability.

It is important to note that the number of training samples before and after augmentation is not fixed but depends on the number of training epochs and the actual application of augmentation strategies during each epoch. The validation and test sets remained unchanged throughout, containing 4893 and 12,452 slices, respectively, to ensure the reliability of evaluation metrics and the reproducibility of the experimental results. The experiment was conducted in a precisely configured hardware and software environment to ensure the reproducibility and reliability of the liver semantic segmentation results. In terms of hardware, the system used the Windows 11 operating system equipped with an Intel Core i7-9700K processor (3.80 GHz) manufactured by Intel Corporation in Santa Clara, CA, United States, an NVIDIA GeForce RTX 2080 Ti high-performance graphics card manufactured by GIGABYTE in New Taipei City, Taiwan, China, and 32 GB of RAM manufactured by Samsung Electronics in Seoul, South Korea to meet the requirements of deep learning model training. The software environment was based on the PyTorch 1.12.1 framework, along with Python 3.7.13, utilizing CUDA 12.0 and cuDNN 8.1 acceleration libraries to optimize GPU performance. The detailed hardware and software environment is shown in Table 4.

In the experiment’s hyperparameter settings, a total of 50 epochs were set for training, with each batch containing 4 samples to balance computational efficiency and memory usage. The learning rate was dynamically adjusted using the cosine annealing strategy, with an initial maximum value of 10^−4^ and a minimum value of 10^−7^, aiming to achieve rapid convergence to an approximate optimal solution before gradually decreasing to prevent overfitting. The Adam optimizer was chosen, widely used in deep learning for its efficient adaptive learning rate adjustment mechanism, which helped the model converge quickly and stably. The specific hyperparameter settings are listed in Table 5.

## 5. Loss Function

In deep learning, the loss function is a critical metric that quantifies the discrepancy between model predictions and ground truth labels, guiding the optimization of model parameters by minimizing this difference.

The liver semantic segmentation task addressed in this study is a foreground–background binary classification problem, aiming to accurately distinguish the liver region from the non-liver region in abdominal CT images. Under this task setting, the liver typically occupies a relatively large anatomical area, and after appropriate preprocessing, the pixel distribution between the foreground (liver) and background tends to be relatively balanced. Given this class distribution, the Binary Cross Entropy Loss (BCE Loss) is capable of achieving effective optimization. BCE Loss calculates the per-pixel probability error with smooth gradient variation, which helps improve stability during both the initial training phase and convergence. In clinical applications, pixel-level classification accuracy is paramount; BCE Loss optimizes predictions at the individual pixel level, thereby meeting the requirements of precise segmentation. Given the proposed model’s strong feature extraction capability to effectively capture both global and local liver information, BCE Loss alone suffices to optimize the segmentation task without the need for more complex loss function combinations. In summary, considering the characteristics of liver segmentation, model adaptability, and training stability, BCE Loss was selected as the optimization objective to ensure robust performance while maintaining a simple and efficient model architecture.

For the task of liver image semantic segmentation, BCE Loss was employed. The formula is as follows:

(28) BCE Loss=−1N ∑i=1N [yi log(pi )+(1−yi )log(1−pi )]

where N represents the total number of pixels in the image; $y_{i}$ denotes the ground truth label, where 1 indicates liver and 0 indicates non-liver; and $p_{i\;}$ is the predicted probability from the model that pixel belongs to the liver class.

By comparing the model’s predicted probabilities $p_{i\;}$ with the ground truth labels $y_{i}$, the BCELoss calculates the cross-entropy for each pixel, quantifying the difference between predictions and true labels. A lower loss value indicates that the model’s predictions are closer to the ground truth. In the context of liver image semantic segmentation, BCELoss is used to train deep learning models to accurately classify each pixel as either liver or non-liver, thereby achieving precise image segmentation.

## 6. Experimental Results and Analysis

### 6.1. Ablation Study Analysis

To evaluate the effectiveness of the proposed TAMU-Net architecture, an ablation study was conducted by selectively removing the MSCA module, ASPP module, and the Channel Transformer module. In this study, the symbols M, A, and T, respectively, denote the MSCA module, the ASPP module, and the Channel Transformer module. Six segmentation metrics were employed to assess the performance, including Precision, Recall, Dice similarity coefficient (Dice), Intersection over Union (IoU), Mean IoU (MIoU), Accuracy, and Mean Pixel Accuracy (MPA). Given that the liver semantic segmentation task in this study is a binary classification problem comprising the liver region and the background, IoU_L_ represents the Intersection over Union for the liver region, IoU_G_ denotes the Intersection over Union for the background, and MIoU indicates the overall segmentation performance, calculated as the average of IoU_L_ and IoU_G_. The detailed results are presented in Table 6.

Compared with TAMU-Net, the variant TAU-Net, which excludes the MSCA module in the encoder, shows a decline of 1.55%, 0.39%, and 1.82% in Precision, Recall, and IoU_L_, respectively. The absence of the MSCA module limits the model’s capability in feature extraction and weakens its ability to recover the fine-grained details lost during downsampling.

The TMU-Net variant, which omits the ASPP module from the bottleneck layer, demonstrates a reduction of 0.75%, 0.19%, and 0.89% in Precision, Recall, and IoU_L_, respectively. This decline indicates a weakened ability to capture global contextual information, which is critical for the accurate segmentation of the liver and surrounding tissues.

In the AMU-Net variant, the Channel Transformer module between the encoder and decoder is replaced with the original skip connection design from U-Net. This results in a decrease of 0.29%, 0.49%, and 0.73% in Precision, Recall, and IoU_L_, respectively. The absence of the Channel Transformer restricts effective cross-scale feature interaction and fails to adequately bridge the semantic gap between shallow and deep layers, thereby limiting feature fusion quality.

Compared with the baseline U-Net, all three variants (TMU-Net, AMU-Net, and TAU-Net) show performance improvements across various metrics. Among them, TAMU-Net consistently achieves the highest performance, demonstrating that the three proposed modules are complementary and mutually reinforcing. The combination of MSCA, ASPP, and Channel Transformer modules significantly enhances segmentation performance and effectively improves the baseline U-Net architecture for liver CT image segmentation.

### 6.2. Analysis of 2D Segmentation Comparative Experiments

To further validate the performance advantages of the proposed liver semantic segmentation method, a comprehensive comparison was conducted between TAMU-Net and six state-of-the-art network architectures: U-Net, Vgg16U-Net, ResNet50U-Net, AttentionU-Net, TransU-Net, and Swin-Unet. Consistent with the ablation study, six segmentation metrics were adopted to evaluate the algorithms’ performance, namely Precision, Recall, Dice similarity coefficient (Dice), Intersection over Union (IoU), Mean IoU (MIoU), Accuracy, and Mean Pixel Accuracy (MPA). The detailed comparison results are summarized in Table 7.

Compared with Vgg16U-Net and ResNet50U-Net, the proposed TAMU-Net achieved improvements of 2.28% and 0.69% in Precision, 0.49% and 1.51% in Recall, and 2.60% and 2.02% in IoU_L_, respectively. Both Vgg16U-Net and ResNet50U-Net enhance the encoder by integrating Vgg16 and ResNet50 backbones to improve the nonlinear representational capacity of feature extraction. However, these networks mainly focus on enhancing feature encoding while overlooking the significant semantic and resolution gaps between different encoder stages. As a result, the conventional skip connections fail to fully bridge the semantic gap and lack effective cross-scale feature integration.

Compared with AttentionU-Net, TAMU-Net showed improvements of 1.63% in Precision, 0.79% in Recall, and 2.26% in IoU_L_. AttentionU-Net introduces spatial attention mechanisms into skip connections, which enhances important feature responses while suppressing irrelevant or noisy information, thereby facilitating feature fusion. However, its performance remains limited due to several factors. The feature extraction capability of the encoder is not substantially improved, which restricts the overall representational capacity of the network. Moreover, the attention mechanism is confined to interactions within the same scale and lacks the ability to model inter-channel dependencies. In addition, the use of simple skip connections does not adequately address the semantic gap between shallow and deep features, thereby hindering the integration of multi-level information.

Compared with TransU-Net and Swin-Unet, TAMU-Net achieved improvements of 1.47% and 5.74% in Precision, 0.80% and 3.00% in Recall, and 2.11% and 7.91% in IoU_L_, respectively. TransU-Net replaces the encoder of U-Net with a Transformer to better capture contextual dependencies, while Swin-Unet introduces a Swin Transformer-based hierarchical feature extraction approach, which divides the input image into non-overlapping patches and performs multi-scale feature fusion. Although both models enhance global feature modeling, they share several limitations. Firstly, the use of standalone skip connections can introduce noise and ambiguous information, which hinders effective semantic alignment. Secondly, neither model explicitly addresses inter-channel relationships, resulting in suboptimal feature interaction and potential semantic gaps.

In contrast, TAMU-Net incorporates three key innovations that address these limitations. The MSCA module in the encoder enhances the model’s ability to extract multi-scale features from complex liver structures. The ASPP module in the bottleneck layer facilitates the capture of rich global contextual information, laying a solid foundation for decoding. Furthermore, the proposed Channel Transformer module replaces conventional skip connections, enabling effective cross-channel feature interaction and fusion between encoder and decoder features. Collectively, these components significantly enhance the overall segmentation performance. In summary, TAMU-Net addresses the deficiencies of the existing models by improving feature extraction, contextual modeling, and cross-scale fusion, thereby advancing liver semantic segmentation performance.

The FLOPs of TAMU-Net amount to 44.10 G, placing it at a moderately high level. Compared to traditional models such as U-Net, Vgg16U-Net, and AttentionU-Net, TAMU-Net exhibits lower computational complexity, indicating the effective optimization of computational resource consumption while maintaining strong feature extraction capabilities. However, when compared with lightweight models like Swin-UNet and ResNet50U-Net, TAMU-Net’s FLOPs are higher, primarily due to the integration of complex attention mechanisms and Transformer modules designed to enhance the modeling of global contextual information. Regarding the number of parameters, TAMU-Net contains 70.66 million, second only to TransU-Net’s 93.23 million, and significantly higher than other models. This parameter scale reflects the extensive use of structural modules such as multi-scale attention mechanisms and Channel Transformer modules to improve the model’s representational capacity, thereby enhancing adaptability to anatomically complex organs like the liver. In contrast, traditional architectures like U-Net and Vgg16U-Net are relatively simple with fewer parameters, making them less capable of meeting the demands of complex tasks.

In summary, TAMU-Net achieves a favorable balance between performance and complexity. Although its parameter size is relatively large, the computational complexity remains manageable and is significantly lower than that of TransU-Net, demonstrating a design focus on balancing efficiency and modeling capability. By incorporating Transformer and multi-scale attention mechanisms, the model substantially improves global information modeling ability, enhancing network performance without substantially increasing computational burden, making it well-suited for high-precision medical image segmentation tasks.

### 6.3. Analysis of 3D Segmentation Comparative Experiments

In medical imaging, computed tomography (CT) provides comprehensive three-dimensional volumetric data, encompassing information along the X, Y, and Z axes. However, conventional two-dimensional semantic segmentation methods are inherently limited to processing individual slices and fail to effectively leverage information along the Z-axis, thereby compromising analytical depth and accuracy. To address this limitation, the present study investigates a strategy to extend 2D segmentation results into 3D space, ensuring the preservation of Z-axis information and enabling more accurate modeling and prediction of three-dimensional anatomical structures. To comprehensively evaluate the performance of various algorithms in 3D semantic segmentation, six key metrics were employed: Dice similarity coefficient (DICE), Intersection over Union (IOU), Volume Overlap Error (VOE), Relative Volume Difference (RVD), Average Symmetric Surface Distance (ASD), and Maximum Symmetric Surface Distance (MSD). The detailed experimental results are presented in Table 8.

In terms of metric interpretation, higher DICE and IOU values indicate better segmentation performance. VOE complements IOU, such that higher IOU corresponds to lower VOE. RVD, ASD, and MSD are distance-based metrics, where lower values indicate more accurate segmentation results.

Compared with six classical segmentation models (U-Net, Vgg16U-Net, ResNet50U-Net, AttentionU-Net, TransU-Net, and Swin-Unet), TAMU-Net achieved superior performance in terms of DICE and IOU scores while also exhibiting lower VOE, RVD, ASD, and MSD values. These results demonstrate the effectiveness of TAMU-Net in liver 3D semantic segmentation and confirm its robustness and generalization capability.

Further improvements in segmentation accuracy not only refine the delineation of liver boundaries but also significantly enhance the precision of liver volume estimation. Liver volume is a critical parameter for various clinical decisions, particularly in surgical planning for hepatectomy, radiotherapy dose design, and postoperative functional assessment. Consequently, high-quality segmentation results can effectively reduce both false negatives and false positives in lesion identification, thereby improving the reliability of quantitative liver analysis. Moreover, the observed performance gains were achieved under conditions of multi-source heterogeneous data, indicating that the proposed method maintains robust generalizability across varying imaging devices, acquisition protocols, and annotation standards. This demonstrates its strong potential for clinical application.

In most publicly available liver segmentation datasets, such as LiTS, SLIVER07, and CHAOS, only a single instance of expert manual annotation is provided as the reference standard. The absence of repeated annotations by multiple experts hampers a comprehensive evaluation of the stability and consistency of automatic segmentation methods in clinical practice. Previous studies have demonstrated that inter-observer variability exists in liver tissue annotation, while intra-observer consistency tends to be relatively high [56,57]. Nevertheless, the accuracy of most current automated methods is still primarily assessed against single-instance expert annotations, and their clinical applicability remains to be validated within a multi-observer agreement framework. In anatomically complex regions, discrepancies between automated segmentation results and expert annotations often reflect variations within a reasonable range of interpretation rather than definitive errors. Therefore, to objectively evaluate the clinical utility of segmentation algorithms, future research should prioritize the development of datasets annotated by multiple experts or incorporate inter-observer and intra-observer consistency metrics as reference baselines for performance assessment, enabling more clinically meaningful comparative analyses.

### 6.4. Heatmap Comparison Between Baseline and Improved Networks

**Figure 9:**
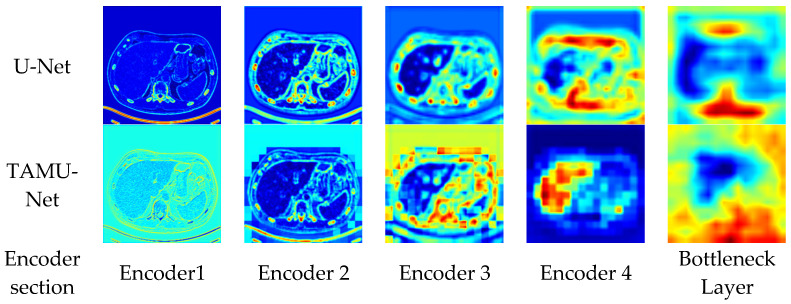
Heatmap comparison between U-Net and TAMU-Net encoders across encoding layers.

**Figure 10:**
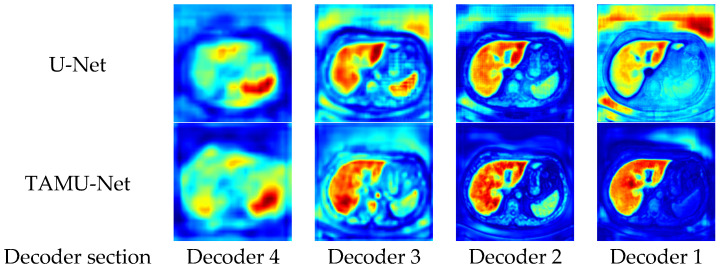
Heatmap comparison between U-Net and TAMU-Net decoders across decoding layers.

To enhance the intuitive understanding and interpretability of the model architecture, this section analyzes the segmentation heatmaps of U-Net and TAMU-Net. Specifically, the first set of heatmaps displays the segmentation results for the U-Net architecture, followed by the corresponding sequence for TAMU-Net. In each set, the first four heatmaps visualize the features extracted at each encoder layer, illustrating the changes in feature extraction as the data undergoes successive downsampling. The fifth heatmap focuses on the bottleneck layer which is the deepest part of the network, showing the abstraction of key features at this stage. The subsequent four heatmaps (columns six to nine) correspond to the decoder part, reflecting the process of progressively recovering spatial resolution and detail during upsampling. By comparing these two sets of heatmaps, a clearer understanding of the differences in feature extraction and reconstruction mechanisms between the two network architectures can be gained when handling image segmentation tasks. The specific visualization results are shown in Figure 9 and Figure 10.

In neural networks, deep and shallow features are extracted from different layers within the network. Shallow features typically arise from the earlier stages of the network, closer to the input layer. These features mainly capture fine-grained information in images, including boundaries, textures, and other local details. In contrast, deep features are extracted from the deeper layers of the network. These features tend to represent coarse-grained information in the image, with higher-level abstraction and semantic understanding, including the overall structure and positional information.

During the downsampling phase, the network progressively learns and extracts features at various levels, from simple local features to more abstract global features, retaining higher-level semantic information at the bottleneck layer. From the downsampling phase’s segmentation heatmaps, TAMU-Net captures richer feature information compared to U-Net. TAMU-Net obtains clearer boundary and texture information in Encoder 1, and richer positional and semantic information in Encoder 4. The capture of richer features during the downsampling phase validates the role of the MSCA module in TAMU-Net, which better extracts both shallow and deep features.

During the upsampling phase, the network synthesizes features learned at different levels from the encoding path to produce more refined image segmentation results. The key in the upsampling process lies in retaining the local and global features extracted from the encoding path and appropriately integrating them through the decoding path to reconstruct the structure and semantic information of the original image.

From the upsampling phase’s segmentation heatmaps, compared to U-Net, TAMU-Net achieves more precise segmentation results as features are progressively passed through the upsampling process. The segmentation areas in decoding Decoder 2 and Decoder 1 are darker, proving TAMU-Net’s focus on the liver region. The precise focus on the segmented regions validates the roles of the Channel Transformer and ASPP modules in TAMU-Net. The ASPP module strengthens the global contextual information, while the Channel Transformer module facilitates the integration of information between decoding and encoding layers, as well as the fusion of features across different levels in the encoding layers.

### 6.5. Comparative Visualization of Segmentation Outcomes

**Figure 11:**
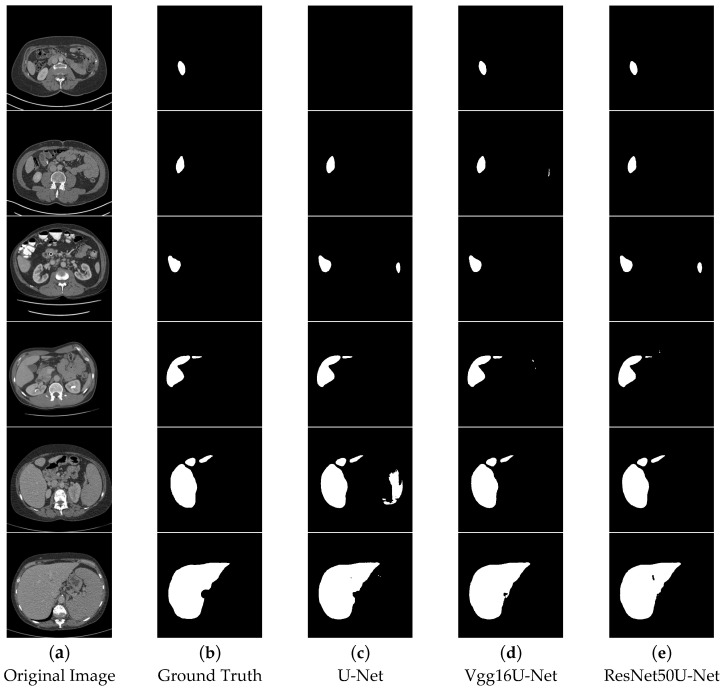
Comparative visualization of segmentation results using different methods.

**Figure 12:**
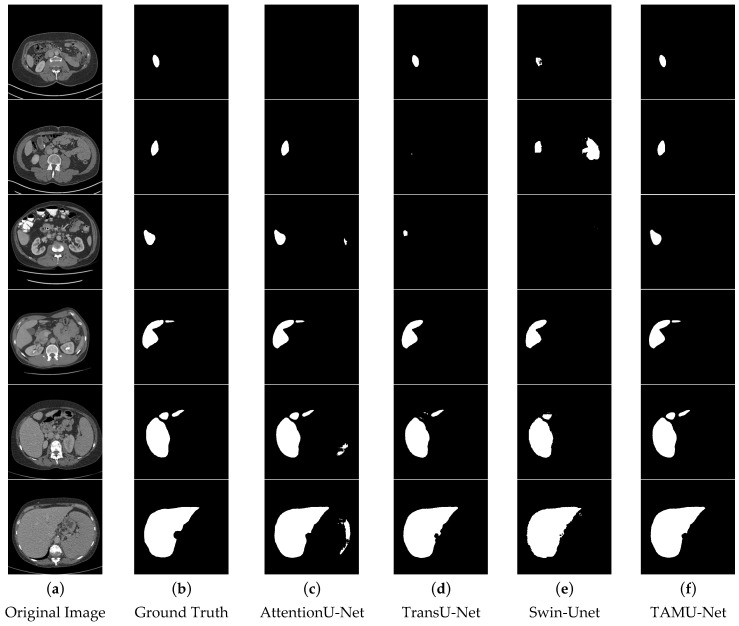
Comparative visualization of segmentation results using different methods.

In the segmentation comparison images, panels a and b in Figure 11 and Figure 12 represent the original CT image and the ground truth, respectively. Panels c, d, and e in Figure 11 show the results of the U-Net, Vgg16U-Net, and ResNet50U-Net models, while c, d, and e in Figure 12 represent the results of the AttentionU-Net, TransU-Net, and Swin-Unet models. Panel f in Figure 12 shows the result of the proposed model, TAMU-Net. The first three rows of segmentation images correspond to small liver regions, the fourth and fifth rows show discontinuous liver regions, and the last row represents larger liver regions. Detailed results are presented in Figure 11 and Figure 12.

All six classic segmentation models (U-Net, Vgg16U-Net, ResNet50U-Net, AttentionU-Net, TransU-Net, and Swin-Unet) exhibit significant issues with over-segmentation and under-segmentation. For small liver regions, the limited size of the liver makes it challenging to distinguish effectively from the background. U-Net, Vgg16U-Net, ResNet50U-Net, AttentionU-Net, and Swin-Unet all suffer from varying degrees of over-segmentation, while U-Net, AttentionU-Net, TransU-Net, and Swin-Unet also show different levels of under-segmentation. For discontinuous liver regions, the irregular shape and position of the liver in the image increase the complexity of the segmentation task. Regarding the segmentation results of four models, U-Net, Vgg16U-Net, ResNet50U-Net, and AttentionU-Net show varying degrees of over-segmentation, while TransU-Net and Swin-Unet exhibit clear under-segmentation. For larger liver regions, AttentionU-Net experiences significant over-segmentation, while the other six models (U-Net, Vgg16U-Net, ResNet50U-Net, AttentionU-Net, TransU-Net, and Swin-Unet) exhibit varying degrees of missing boundary information, including over-segmentation and under-segmentation.

TAMU-Net demonstrates significant advantages in liver image segmentation compared to the other six models, effectively avoiding over-segmentation and under-segmentation. For small regions, TAMU-Net can identify and segment details that are difficult to discern by the human eye, showcasing its outstanding segmentation performance. For discontinuous regions, TAMU-Net accurately identifies and segments areas with irregular shapes and positions, demonstrating its adaptability to complex shapes and discontinuous locations. Furthermore, for larger regions, TAMU-Net can identify and segment areas with relatively regular shapes and positions, yet potentially complex backgrounds, highlighting its capability in handling large regions and complex backgrounds. TAMU-Net’s superiority lies in three key aspects: (1) the new model incorporates the MSCA module in the encoder, enhancing the feature extraction ability; (2) the Channel Transformer module replaces the original skip connections, strengthening effective feature interaction, reducing the semantic gap, and promoting the fusion of deep and shallow features; (3) the ASPP structure strengthens the acquisition of global contextual information.

### 6.6. Comparative Analysis of Segmentation Heatmaps

**Figure 13:**
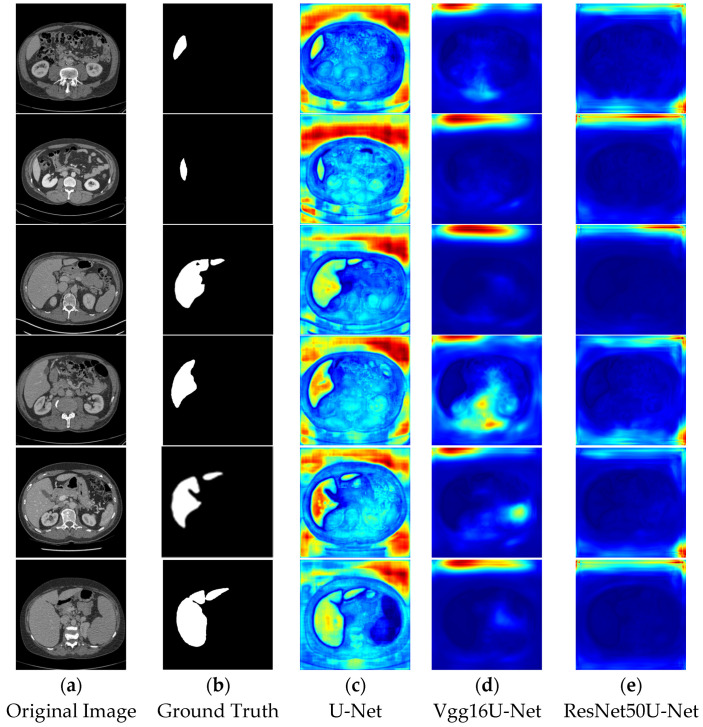
Comparative visualization of heatmaps using different methods.

**Figure 14:**
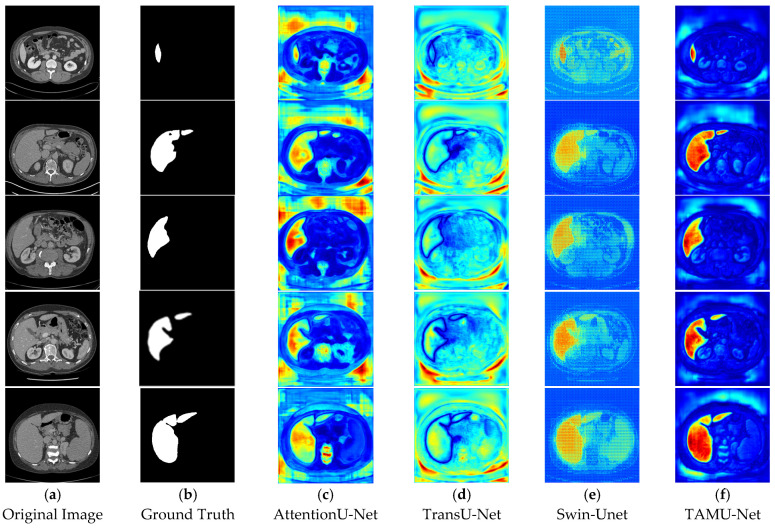
Comparative visualization of heatmaps using different methods.

In the segmentation comparison heatmaps, panels a and b in Figure 13 and Figure 14 represent the original CT image and the ground truth, which serves as the reference for evaluating model performance. Panels c, d, and e in Figure 13 represent the U-Net, Vgg16U-Net, and ResNet50U-Net models, respectively, while Panels c, d, and e in Figure 14 show the AttentionU-Net, TransU-Net, and Swin-Unet models. Panel f in Figure 14 shows the result of the proposed TAMU-Net model. The results represent the segmentation heatmaps of the final layer of the decoder. Detailed results are shown in Figure 13 and Figure 14.

When comparing the impact of different network architectures on image segmentation tasks, we found that the segmentation results of Vgg16U-Net and ResNet50U-Net lack clear region boundaries. This may be attributed to their insufficient learning and capture of key image features and contextual information. In contrast, U-Net and its variants (AttentionU-Net and TransU-Net), by incorporating mechanisms such as attention mechanisms and Transformer structures, have enhanced feature processing. However, the segmentation results still show uneven color distribution and boundary handling deficiencies, indicating that these models still have room for improvement in balancing the boundary delineation of different classes. Specifically, Swin-Unet, due to its unique sliding window mechanism, produces a grid-like effect in the segmentation results, and the segmented regions have less pronounced colors. This likely reflects the model’s uncertainty and smoothing effects in the feature-to-class mapping process, further highlighting its limitations in handling fine image boundary details.

In contrast, the TAMU-Net model shows the strongest focus on the segmented regions, which appear in bright red. This indicates that TAMU-Net outperforms other network architectures in terms of segmentation performance. TAMU-Net combines the MSCA module, Channel Transformer module, and ASPP module, allowing it to better capture contextual information and local details in the image, and improve the accuracy of boundary delineation between different classes. Overall, by comparing the segmentation results of various models, it is clear that TAMU-Net delivers the best performance in semantic segmentation tasks.

## 7. Conclusions

This paper proposes a liver CT image semantic segmentation method based on attention mechanisms and multi-scale feature fusion (TAMU-Net), aiming to address the issues of insufficient feature extraction, inadequate multi-scale information fusion, and lack of global feature perception in liver segmentation. By introducing the MSCA module in the encoding part, the model enhances the encoder’s ability to extract liver features. The ASPP module is used in the bottleneck layer to improve the acquisition of high-level semantic information. Additionally, the Channel Transformer module replaces traditional skip connections, effectively bridging the semantic gap and promoting the deep fusion of shallow and deep features. This paper combines the LiTS, SLIVER07, and CHAOS datasets for the first time and validates the method’s accuracy on the combined dataset. Compared to mainstream semantic segmentation methods, TAMU-Net achieves superior results in segmentation metrics. In the ablation experiments, the method achieved the best metrics, demonstrating the effectiveness of the network architecture. Moreover, the paper retains the Z-axis information and extends the 2D semantic segmentation results to 3D, further valid.

The introduction of TAMU-Net holds significant theoretical and practical value in the field of liver segmentation. Firstly, by introducing a multi-scale feature extraction mechanism, the method significantly enhances the model’s ability to represent complex liver structures, providing a technical guarantee for high-precision segmentation. Secondly, the design of the new channel transformation mechanism effectively addresses the insufficient fusion of shallow and deep features in traditional methods, enhancing the consistency and stability of the segmentation results. Furthermore, TAMU-Net’s outstanding performance on the combined dataset validates its strong generalization ability, providing reliable support for the application of liver segmentation technology in various clinical scenarios. Finally, by extending 2D segmentation to 3D, the method not only improves segmentation accuracy but also provides new insights for 3D medical image analysis, advancing liver segmentation technology toward higher-dimensional applications.

Although the proposed model demonstrates notable improvements in liver segmentation accuracy and generalization capability, several challenges remain that warrant further investigation and optimization:

(1) The integration of MSCA, ASPP, and Channel Transformer modules increases the structural complexity of the network, resulting in a larger number of parameters and higher computational overhead. Consequently, the model imposes substantial demands on memory and inference speed, limiting its deployment in resource-constrained or real-time applications.

(2) The current experiments are primarily conducted on publicly available multi-source datasets, which encompass data from diverse institutions and imaging devices. However, further validation using large-scale, representative real-world clinical data is necessary to assess the model’s robustness and generalizability in the presence of complex lesion characteristics and inter-patient variability.

(3) While the hybrid data augmentation strategy enhances model performance, its effectiveness on cross-modal images (e.g., ultrasound and MRI) and on low-quality or high-noise CT scans has not been systematically evaluated, limiting its applicability to diverse clinical scenarios.

To further improve the practicality and clinical viability of the proposed method, future research may focus on the following directions:

(1) Emphasize model lightweighting and efficient inference techniques by incorporating strategies such as model pruning, parameter quantization, and knowledge distillation, thereby reducing computational complexity and resource consumption to meet the requirements of mobile and edge devices.

(2) Enhance external validation by conducting systematic evaluations on larger-scale, multi-center clinical datasets involving various diseases to assess the model’s adaptability and stability across different imaging protocols, devices, and lesion characteristics.

(3) Further expand investigations into the model’s adaptability to cross-modal and low-quality images by integrating modality transfer learning, universal feature extraction architectures, and robustness enhancement strategies, thus improving performance in heterogeneous imaging environments.

(4) In parallel, establish standardized protocols for data processing and usage to ensure quality control, privacy protection, and information security during model training and deployment, thereby laying a solid foundation for stable integration into real-world clinical systems.

## Figures and Tables

**Table 1:** Liver disease detection systems and applications of artificial intelligence.

Disease Type	Liver Segmentation Required	CT Imaging Required	Integration with Other Biosensors	Application of AI Systems
Primary Hepatocellular Carcinoma (HCC) [6]	Yes	Yes	Optional (e.g., AFP and ctDNA)	Lesion detection, preoperative assessment, prognosis prediction, tumor volume, and burden estimation
Intrahepatic Cholangiocarcinoma (ICC) [7]	Yes	Yes	Optional (e.g., CA19-9 and MRI)	Lesion identification, surgical planning, volumetric, and liver function assessment
Liver Metastases [8]	Yes	Yes	Optional (e.g., PET-CT, CEA, CA125, or markers of primary tumors)	Multifocal lesion detection, treatment monitoring, and liver volume change evaluation
Liver Cirrhosis [9]	Yes	Yes	Optional (e.g., elastography and serological markers)	Complication prediction, rejection monitoring, and evaluation of graft liver volume and function
Post-Liver Transplantation Management [10]	Yes	Yes	Yes (e.g., liver function markers and immune rejection indicators)	Complication prediction, rejection surveillance, and evaluation of graft liver volume and function

**Table 2:** Comparison of technical advantages, limitations, and application suitability.

Category	Representative Models	Advantages	Limitations	Applicable Scenarios
Traditional Methods	Level Set, Thresholding, and Region Growing	Simple algorithms, highly interpretable, and easy to implement	Relies on manually set parameters, low robustness, and struggles with complex structures and image variability	Applications with simple anatomical structures or real-time processing requirements
Pure CNN-based Methods	FCN, SegNet, and U-Net	Strong local feature extraction, relatively simple architecture, and stable training	Limited receptive field, poor modeling of long-range dependencies, and lacks global contextual understanding	Segmentation tasks involving regular anatomical structures and single imaging modalities
Improved U-Net Variants	ResU-Net, Dense-PSP-UNet, and U-Net++	Incorporates multi-scale information, enhanced detail capture, and high accuracy	Limited feature transmission, insufficient fusion, potential redundancy, and still lacks global modeling ability	Medical image segmentation requiring fine-grained boundary delineation or complex organ shapes
Attention-Enhanced Models	ERes-UNet++, EAR-U-Net, and MSA-UNet	Emphasizes critical regions, suppresses background noise, and improves discrimination	Limited cross-layer feature fusion, attention mechanism may introduce errors, and lacks full global context	Tasks with noisy backgrounds requiring precise localization of key regions
CNN-Transformer Hybrid Models	TransU-Net, Swin-Unet, and ST-Unet	Combines local feature extraction with global context modeling	Significant semantic gap between CNN and Transformer features, challenging integration, and complex architecture	Complex medical image segmentation tasks requiring both local precision and global understanding
Federated Learning Methods	Variants of Federated U-Net	Enables privacy protection and improves generalizability across multi-center data	High communication costs, large domain shifts across datasets, and model aggregation may be affected	Multi-center collaborative modeling, scenarios with strict data privacy or regulatory requirements
Explainable AI Approaches	Grad-CAM and Integrated XAI Modules	Improves model transparency and trustworthiness	Interpretation depends on model structure and limited insight into internal mechanisms	Clinical decision support, tasks requiring high credibility and interpretability of results

**Table 3:** Dataset partitioning summary.

Dataset	Training Set	Validation Set	Test Set
LiTS	104	13	14
SLIVER07	16	2	2
CHAOS	16	2	2
Total	136	17	18

**Table 4:** Hardware and software configuration.

Environment	Configuration
Operating System	Windows 11
Processor	Intel^®^ Core™ i7-9700K CPU @ 3.80 GHz
GPU	NVIDIA GeForce RTX 2080 Ti
Memory	32 GB
Experimental Platform	PyTorch 1.12.1, Python 3.7.13, CUDA 12.0, cuDNN 8.1

**Table 5:** Hyperparameter settings.

Hyperparameter Type	Configuration
Epochs	50
Batch Size	4
Maximum Learning Rate	1 × 10^−4^
Minimum Learning Rate	1 × 10^−7^
Learning Rate Scheduler	Cosine Annealing
Optimizer	Adam
Loss Function	Binary Cross Entropy

**Table 6:** Ablation experiment results of TAMU-Net.

Model	Precision	Recall	IoU_L_	IoU_G_	MIoU	Accuracy	MPA	Dice
U-Net	0.9342	0.9673	0.9056	0.9976	0.9516	0.9977	0.9829	0.9504
TAU-Net	0.9398	0.9700	0.9133	0.9979	0.9556	0.9979	0.9843	0.9548
TMU-Net	0.9478	0.9720	0.9226	0.9982	0.9604	0.9981	0.9854	0.9599
AMU-Net	0.9524	0.9690	0.9242	0.9982	0.9612	0.9982	0.9839	0.9606
TAMU-Net	0.9553	0.9739	0.9315	0.9983	0.9649	0.9984	0.9864	0.9646

**Table 7:** Segmentation results of mainstream models.

Model	Precision	Recall	IoU_L_	IoU_G_	MIoU	Accuracy	MPA	Dice	FLOPs	Parameters
U-Net	0.9342	0.9673	0.9056	0.9976	0.9516	0.9977	0.9829	0.9504	54.82 G	31.04 M
Vgg16U-Net	0.9325	0.9690	0.9055	0.9977	0.9516	0.9977	0.9837	0.9503	56.46 G	24.89 M
ResNet50U-Net	0.9484	0.9588	0.9113	0.9977	0.9545	0.9979	0.9788	0.9539	23.01 G	43.93 M
AttentionU-Net	0.9390	0.9660	0.9089	0.9977	0.9533	0.9978	0.9822	0.9525	66.63 G	34.88 M
TransU-Net	0.9406	0.9659	0.9104	0.9978	0.9541	0.9978	0.9822	0.9534	32.42 G	93.23 M
Swin-Unet	0.8979	0.9439	0.8524	0.9962	0.9243	0.9963	0.9709	0.9204	14.01 G	27.24 M
TAMU-Net	0.9553	0.9739	0.9315	0.9983	0.9649	0.9984	0.9864	0.9646	44.10 G	70.66 M

**Table 8:** Comparison of 3D metrics for mainstream models.

Model	DICE	IOU	VOE	RVD	ASD	MSD
U-Net_3D_	0.9612	0.9254	0.0746	0.0496	4.9507	217.2694
Vgg16U-Net_3D_	0.9417	0.8914	0.1086	0.0892	25.2042	285.6919
ResNet50U-Net_3D_	0.9409	0.8899	0.1101	0.0855	20.7555	251.0137
AttentionU-Net_3D_	0.9677	0.9377	0.0623	0.0321	6.3682	208.9805
TransU-Net_3D_	0.9567	0.9177	0.0823	0.0267	10.4802	228.7672
Swin-Unet_3D_	0.8445	0.7392	0.2608	0.0727	31.5956	253.3908
TAMU-Net_3D_	0.9715	0.9446	0.0554	0.0188	4.3027	187.1961

## Data Availability

No new data were created or analyzed in this study. The original contributions presented in the study are included in the article, further inquiries can be directed to the corresponding author.
